# A Comparative in Silico Analysis of CD24’s Prognostic Value in Human and Canine Prostate Cancer

**DOI:** 10.3390/jpm11030232

**Published:** 2021-03-23

**Authors:** Antonio Fernando Leis-Filho, Patrícia de Faria Lainetti, Mayara Simão Franzoni, Chiara Palmieri, Priscila Emiko Kobayshi, Renee Laufer-Amorim, Carlos Eduardo Fonseca-Alves

**Affiliations:** 1 Department of Veterinary Surgery and Animal Reproduction, Sao Paulo State University-UNESP, Botucatu 18618-681, Brazil; a.leis@unesp.br (A.F.L.-F.); patricia.lainetti@unesp.br (P.d.F.L.); mayara.s.franzoni@unesp.br (M.S.F.); 2 School of Veterinary Science, Gatton Campus, The University of Queensland-UQ, Brisbane Qld 4343, Australia; c.palmieri@uq.edu.au; 3 Department of Veterinary Clinic, Sao Paulo State University-UNESP, Botucatu 18618-681, Brazil; priscila.e.kobayashi@unesp.br (P.E.K.); renee.laufer-amorim@unesp.br (R.L.-A.); 4 Laboratório de Patologia, Faculdade de Ensino Superior e Formação Integral-FAEF, Garça 17400-000, Brazil; 5 Institute of Health Sciences, Paulista University–UNIP, Bauru 18618-681, Brazil

**Keywords:** carcinoma, prostate, CD24, comparative oncology, dog

## Abstract

CD24 is a cell surface molecule anchored by glycosyl-phosphatidyl-inositol and expressed by different human cancers, including prostate cancer (PC). Some studies have demonstrated that CD24 expression is associated with poor patient outcome; however, few studies have investigated CD24 expression in spontaneous animal models of human PC, such as canine PC. This study aimed to evaluate the expression of CD24 in human PC using the in silico analysis of the data obtained from The Cancer Genome Atlas (TCGA) and comparing it with the previously published prostatic canine transcriptome data. In addition, CD24 expression was confirmed by immunohistochemistry in an independent cohort of canine prostatic samples and its prognostic significance assessed. The systematic review identified 10 publications fitting with the inclusion criteria of this study. Of the 10 manuscripts, 5 demonstrated a direct correlation between CD24 overexpression and patient prognoses. CD24 expression was also associated with PSA relapse (2/5) and tumor progression (1/5). However, the in silico analysis did not validate CD24 as a prognostic factor of human PC. Regarding canine PC, 10 out of 30 normal prostates and 27 out of 40 PC samples were positive for CD24. As in humans, there was no association with overall survival. Overall, our results demonstrated a significant CD24 overexpression in human and canine prostate cancer, although its prognostic value may be questionable. However, tumors overexpressing CD24 may be a reliable model for new target therapies and dogs could be used of a unique preclinical model for these studies.

## 1. Introduction

CD24 is an adhesion molecule anchored by glycosyl-phosphotidyl-inositol and dysregulated in several cancer subtypes, including renal, breast, ovarian, lung, and pancreatic cancers [1,2]. CD24 was recently investigated as a potential cancer stem cell (CSC) marker [1,3,4], although the results were ambiguous. CD24 has been evaluated as a stem cell marker in association with CD44. Few cancer cell lines have been actually classified as being CD44-positive and CD24-negative (CD44^+^/CD24^−^) [1,3,4], thus questioning the significance of CD24 as a CSC surface marker. However, its key role in tumor progression and metastasis is well recognized, in particular in breast, colorectal, and liver cancers [5], and recently as a prognostic factor for prostate cancer (PC) [6,7,8,9].

In addition to its importance as a prognostic biomarker, CD24 is considered a target for anti-cancer therapies [10,11]. The exact functions of CD24 during oncogenesis are not completely understood [11]. Recent evidence has demonstrated the role of CD24 upregulation in the activation of different tyrosine and serine pathways, including MAPK, EFGR, STAT3 and Wnt/β-catenin [11]. CD24 has also been associated with immune evasion through selective binding to macrophage molecules [10]. Interestingly, one preclinical model targeting CD24 has been developed, a nanoparticle targeting CD24-positive cells and anti-CD24 monoclonal antibodies [12,13,14].

The new recent concepts on CD24 open interesting perspectives for innovative cancer treatments. However, these studies are in the initial stage of development and the evaluation of the efficacy of these new therapies in in vivo preclinical models are pivotal for their application in human clinical practice [15,16]. In this scenario, dogs represent a unique model for cancer research. Dogs are considered the only species, other than humans, that develop spontaneous PC, with similar aggressive behavior and response to treatment and histological patterns [17]. On the other hand, little is known about the role of CD24 in canine PC, making any possible comparative applicability of potential CD24-focused cancer therapies difficult. Only two previous studies investigated the expression of CD24 in canine PC [3,4]. However, these studies were focused on regions of the tumors negative for CD24, as a stem cell marker, and no information regarding the actual positive expression of CD24 was provided. Thus, this study aims to perform a systematic review and in silico analysis of CD24 to investigate its prognostic value in human and canine PC.

## 2. Materials and Methods

### 2.1. Study Design

**Figure 1:**
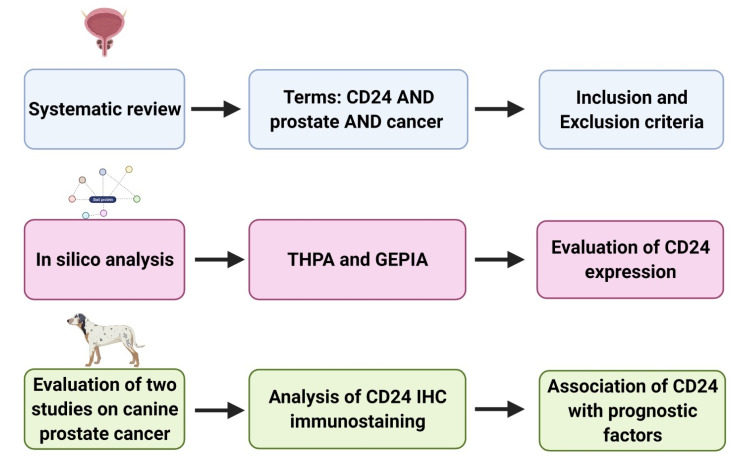
Schematic representation of the study design. (1) Systematic review of the previous literature and selection of manuscripts evaluating prognostic significance of CD24 in human prostate cancer. (2) In silico analysis to evaluate CD24 expression in prostate cancer compared to normal prostate (as found by Gene Expression Profiling Interactive Analysis (GEPIA)) and association between CD24 and patients’ survival (GEPIA and The Human Protein Atlas (THPA)). (3) Retrieval of CD24 immunohistochemistry slides from two previous veterinary studies and re-evaluation of the CD24 expression in association with clinical pathological characteristics. Figure generated in BioRender (www.biorender.com, accessed on 15 November 2020).

This study consisted of two steps as summarized in Figure 1. A preliminary systematic review was performed to identify previous studies evaluating CD24 expression in human prostate cancer. All the available information was retrieved and analyzed. The systematic review was followed by an in silico analysis, considering data available in the Gene Expression Profiling Interactive Analysis (GEPIA) (http://gepia.cancer-pku.cn/, accessed on 15 November 2020) [18] and The Human Protein Atlas (THPA) project (https://www.proteinatlas.org/, accessed on 15 November 2020) [5] in order to identify the correlation between CD24 expression and patient prognosis. Finally, information regarding CD24 expression in canine PC was mined from two previous publications [3,4] and the data were analyzed to evaluate the prognostic value of CD24 in the canine spontaneous PC.

### 2.2. Systematic Review

The systematic review was performed according to Gambini et al. [19]. Briefly, the initial search was performed on the PubMed, MEDLINE, and Scielo databases using the following indexing terms “CD24” AND “prostate” AND “cancer” with no restrictions regarding the year of publication. Then, the reference list of the selected manuscripts was evaluated and manually checked for additional publications. Moreover, the most relevant journals on prostate biology and/or pathology were screened to ensure the inclusion of the highest number of relevant manuscripts. Afterwards, the title and the abstract of all the manuscripts were evaluated to select original contributions only, focused on *CD24* gene or protein expression in PC. Manuscripts evaluating CD24 expression in prostate tumor samples or cell lines and focused on the association of CD24 with tumorigenesis, cancer progression, prognosis, or predictive value were included. Manuscripts involving prostate cancer models that met the criteria as described above were also included. Reviews, case reports, and journal articles/research papers evaluating in silico data only were excluded, as well as manuscripts considering only CD24 negative expression, as a cancer stem cell marker. The manuscript content was then analyzed to confirm that every paper was actually evaluating CD24 expression in human PC samples or cell lines and all available clinical and pathological information were retrieved. The first search was performed on 12 September 2020, then updated on 13 November 2020.

### 2.3. In Silico Analysis

To confirm CD24 overexpression in human PC, we analyzed the expression of CD24 in PC samples from The Cancer Genome Atlas (TCGA) (*n* = 492) compared to normal prostate samples from the same database and the Genotype-Tissue Expression (GTEx) project (https://gtexportal.org/home/, accessed on 15 November 2020) matched (*n* = 152), using the Gene Expression Profiling Interactive Analysis (GEPIA) (http://gepia.cancer-pku.cn/, accessed on 15 November 2020) [18]. The survival analysis was performed using the dataset containing 492 prostate cancer (PRAD) samples available in both GEPIA and THPA portals. Using GEPIA, the survival analysis was performed using the CD24 median expression as a cut-off to classify patients as CD24-high and CD24-low. The confidence interval was set to 95% and the axis units were provided in months. We also calculated the disease-free interval using the same parameters. Using the THPA (https://www.proteinatlas.org/, accessed on 15 November 2020) [5], we evaluated the CD24 immunohistochemical expression pattern in normal and PC samples, as well as the association between CD24 and survival. For the immunohistochemical analysis, staining was considered negative, low, moderate, or high according to the information provided by the THPA. A similar criterion for evaluating the association of CD24 and patient survival as described for GEPIA was applied to the second database. In the THPA database, the information as described above were available from 494 patients.

### 2.4. CD24 Expression in Canine PC

Since dogs are considered unique models for human PC studies, we retrieved clinical and pathological information from two studies previously published by our research group [3,4], as well as the corresponding CD24-stained sections to quantify the CD24 positive areas. From the Fonseca-Alves et al. (2018) publication, a total of 48 slides (28 PC and 20 normal prostates) were retrieved, while 22 samples (12 Pc and 10 normal prostate) were obtained from Costa et al. [4]. The immunohistochemical re-evaluation was performed according to the methodology as described by Kristiansen et al. [6], Kristiansen et al. [20], Liu et al. [9]. Briefly, samples showing 10% or less of positive cells were considered negative and samples showing more than 10% of positive cells were considered positive. 

### 2.5. Evaluation of CD24 as a Prognostic Factor in Canine PC

The histological subtype, Gleason score, and overall survival of canine PC were compared with the CD24 expression (positive or negative) to evaluate the correlation between CD24 and prognosis. The association of CD24 expression with the Gleason score and the histological subtype was evaluated using the Fisher exact test. The association of CD24 with overall survival was analyzed using the Kaplan–Meier curves. All the statistical analyses were performed using Graph Pad Prism 8.0.

### 2.6. Comparative Homology and CD24 Expression

The *CD24* gene homology between human and dogs was evaluated using the BLAST online tool from the National Center for Biotechnology Information (NCBI) (https://blast.ncbi.nlm.nih.gov/Blast.cgi, accessed on 15 February 2021) [21]. Briefly, the Genome GRCh38.p13 Reference Annotation 109.20200228 from the human genome was compared to the *CD24* (NCBI Reference Sequence: NC_051816.1) canine gene sequences. Afterwards, a tridimensional protein structure homology-modeling was performed using the Swiss model online tool (https://swissmodel.expasy.org/, accessed on 15 February 2021) [22] and the protein homology of the amino acid sequence of human (NCBI Reference Sequence: XP_024302061.1) and canine (NCBI Reference Sequence: XP_038540218.1) CD24 protein were performed using the NCBI BLAST online tool.

The canine prostate cancer cell lines (PC1 and PC2) and the human prostate cancer cell line (PC3) were used to investigate CD24 expression by immunofluorescence. The canine PC1 and PC2 cell lines were previously established and characterized by our research group [4] and the human prostate PC3 cell line was retrieved from the European Collection of Authenticated Cell Cultures (ECACC). The canine cell lines were used at passage 20 and the human PC3 cell line was used at passage 17. The immunofluorescence procedures were performed according to the previous literature [4]. Briefly, round sterile 1.5 coverslips were placed on 12-well plates (Sigma, Portland, OR, USA) at 1×10^5^ cell density. Then, 250 μL of culture medium (DMEM F12, Lonza, Basel, Switzerland) supplemented with 10% FBS containing the cells were incubated in a humidified atmosphere of 5% CO 2 at 37 °C. The mouse monoclonal CD24 antibody (Santa Cruz Biotechnology, Dallas, TX, USA) was used as primary antibody and the rabbit anti-mouse Alexa Fluor 594 (BioLegend, San Diego, CA, USA) was applied at a 1.5 μg/mL dilution in PBS for 60 min as a secondary antibody. The slides were counterstained with 4 ′-6-diamidino-2-phenylindole (DAPI; Sigma, Portland, OR, USA) and visualized on a confocal microscope (Leica, Wetzlar, Germany).

## 3. Results

### 3.1. Systematic Review

Seventy-eight manuscripts on CD24 expression in human prostate cancer were identified in the original search. After evaluating titles and abstracts, 61 manuscripts not meeting the inclusion criteria were excluded. An additional 7 publications meeting the exclusion criteria were removed, with a final number of 10 manuscripts included in the systematic review. After evaluation of the 10 manuscripts, all available data were retrieved (Table 1). Of the 10 manuscripts, 5 demonstrated a direct relationship between CD24 overexpression and patients’ prognosis (Table 1). In addition, CD24 expression was associated with serum prostatic specific antigen (PSA) relapse (2/5) and tumor progression (1/5).

### 3.2. In Silico Analysis

**Figure 2:**
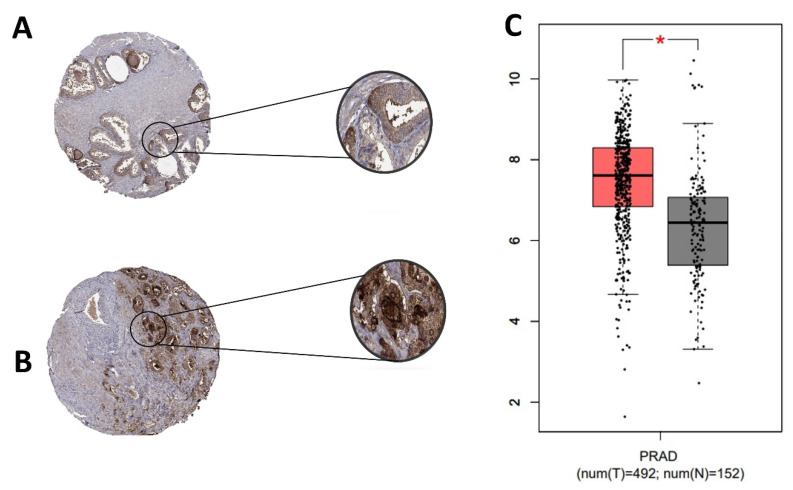
CD24 gene and protein expression in human prostate cancer. (**A**): Tissue microarray (TMA) core from a normal human prostate gland. Normal glandular cells show moderate membranous and cytoplasmic expression. (**B**): A TMA core from a human prostate cancer, with a strong CD24 expression in the membrane and cytoplasm of neoplastic cells. (**C**): CD24 overexpression in prostate cancer samples compared to normal samples (* *p* > 0.01). Image credit of the immunohistochemistry images: Human Protein Atlas, www.proteinatlas.org. Image available at the following URL: https://v20.proteinatlas.org/ENSG00000272398-CD24/tissue (accessed on 15 November 2020). The gene expression dotplot was generated using GEPIA database (http://gepia.cancer-pku.cn/about.html, accessed on 15 November 2020). PRAD: Prostate cancer dataset.

**Figure 3:**
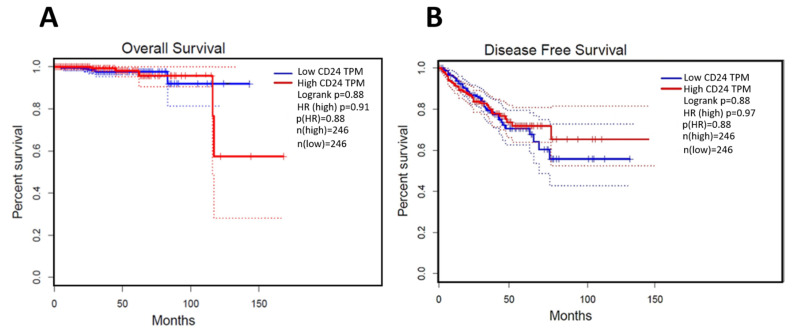
Survival analysis of human patients according to CD24 expression. (**A**): CD24 expression is not associated with overall survival of human prostate cancer patients. (**B**): the disease-free interval is not associated with CD24 expression. The survival curves were generated using the GEPIA database (http://gepia.cancer-pku.cn/about.html, accessed on 15 November 2020).

**Figure 4:**
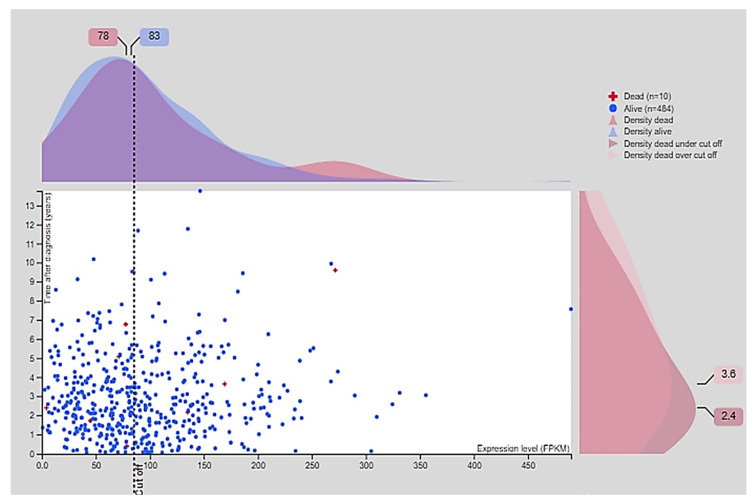
Survival association according to the CD24 fragments per kilobase of exon model per million reads mapped (FPKM). The median of CD24 FPKM in the group of dead human patients is 78 and in the group of living human patients is 83. There is no significant association between CD24 expression and overall survival (*p* > 0.05). Image credit: Human Protein Atlas, www.proteinatlas.org accessed on 15 November 2020. Image available at the following URL: https://v20.proteinatlas.org/ENSG00000272398-CD24/pathology/prostate+cancer#, accessed on 15 November 2020.

In normal samples, CD24 was expressed on the cell membrane and cytoplasm of the glandular epithelial cells with a moderate intensity of staining (Figure 2). In PC samples, CD24 expression was also membranous and cytoplasmic, with an expression pattern ranging from low to high (Figure 2). Our in silico analysis revealed a higher *CD24* gene expression in PC compared to normal samples (*p* > 0.01) (Figure 2). The survival analysis did not find any association between CD24 expression and patients’ overall survival. Using the GEPIA database, no associations were observed between CD24 low or high expression and survival or disease-free interval (*p* = 0.88) (Figure 3). A similar lack of association was observed even considering the survival data evaluated from THPA (*p* > 0.05) with the median CD24 expression in dead patients being 78.1 FPKM (Fragments Per kilobase of transcript per million mapped reads) and in living patients being 83 FPKM (Figure 4).

### 3.3. CD24 Expression in Canine Prostate Samples

**Figure 5:**
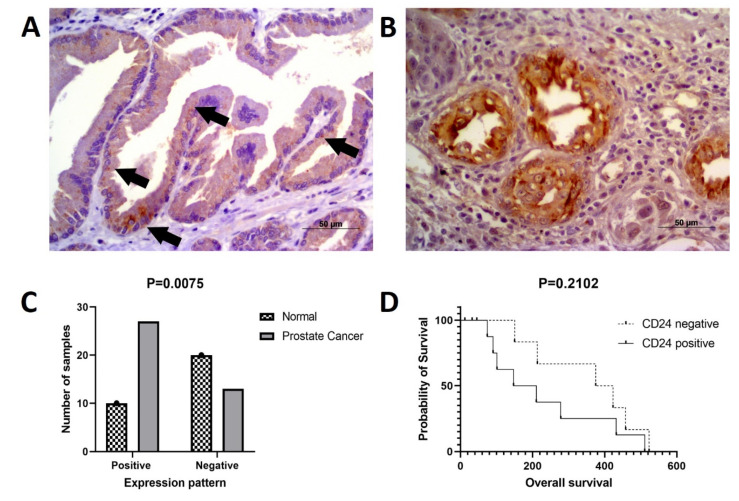
CD24 immunoexpression in canine prostate cancer. (**A**): multifocal cytoplasmic CD24 expression (arrows) in a normal prostate considered positive for CD24. (**B**): canine prostate cancer showing diffuse cytoplasmic and membranous CD24 expression by neoplastic cells. (**C**): CD24 overexpression in canine prostate cancer compared to normal prostate. (**D**): Survival analysis according to CD24 expression. Dogs with CD24 positive expression showed a tendency to lower survival compared to dogs with CD24 negative expression.

Regarding CD24 expression, 10 out of 30 normal prostates were positive for CD24 (Figure 5A). Out of 40 PC samples, 27 positive (Figure 5B) and 13 negative samples were identified. CD24 was overexpressed in PC compared to normal prostate (*p* = 0.0075) (Figure 5C). The association between clinical and pathological findings and CD24 expression is shown in Table 2.

A lower mean survival was observed in canine patients with CD24 positive samples compared to CD24 negative cases, although without any statistical difference (*p* = 0.2102) (Figure 5D). Neither was any association identified between CD24 expression and presence of metastasis at the time of diagnosis (*p* > 0.9999 histological pattern (*p* = 0.4849) and Gleason score (*p* = 0.8420).

### 3.4. Comparative Homology and CD24 Expression

**Figure 6:**
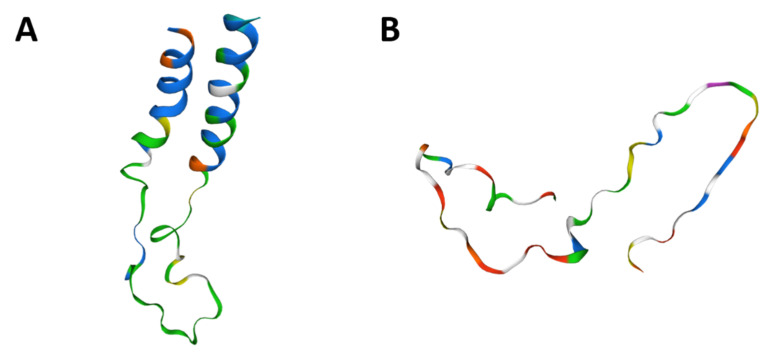
CD24 tertiary protein structure in dogs (**A**) and humans (**B**). Human and canine CD24 proteins showed a 90% similarity among amino acid sequence and also similar tyrosine kinase domains. Figures generated in Swiss model online tool (https://swissmodel.expasy.org/, accessed on 15 November 2020).

**Figure 7:**
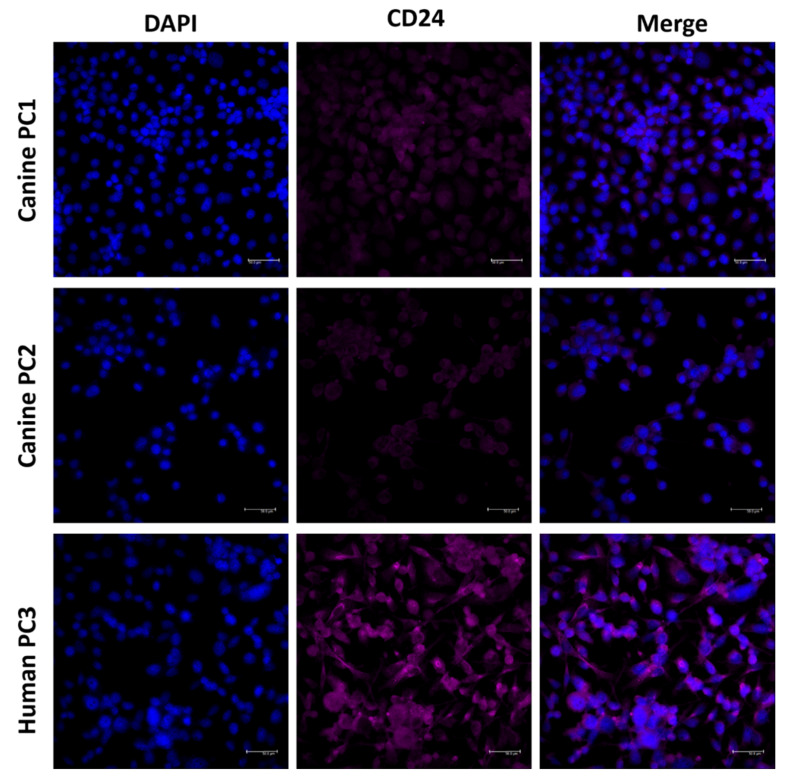
CD24 immunofluorescence on canine (PC1 and PC2) and human (PC3) prostate cancer cell lines. CD24 positive expression was observed in both canine and human cells, demonstrating the potential of these cells to be preclinical models for CD24-targeting drugs.

The *CD24* gene showed 76.25% homology between human and canine sequences. CD24 amino acid sequence and protein structure homology-modeling had a high identity (90%) between humans and dogs. The 90% homology between CD24 canine and human protein included Glycine, Proline, and Pre-Proline residues (Figure 6). CD24 showed positive cytoplasmic expression in both canine cells (PC1 and PC2) and the human prostate cancer PC3 cell line (Figure 7).

## 4. Discussion

In this study, we investigated the potential prognostic value of CD24 in prostate cancer through a systematic review of the previous literature on the expression of CD24 in human PC [7,9,20,24]. The systematic review included studies evaluating the association of CD24 with tumor initiation, progression, and patient prognosis [7,9,20,24]. In 5 publications, the prognostic potential of CD24 was associated with the expression of the same protein by immunohistochemistry [6,8,9,20]. However, in our in silico validation, this prognostic significance could not be confirmed. The discrepancy is likely due to the different methodology used for detecting the expression of CD24. Our study was based on CD24 transcripts obtained through large-scale transcriptome data (mRNA expression), while previous papers included immunohistochemical data.

In the evaluation of CD24 immunoexpression of canine prostate samples, a tendency for a higher survival time was observed in patients with CD24-negative prostate cancer (hazard ratio: 2.235). However, no statistical differences were found. Even performing a different sample segregation (classifying samples as negative, low, moderate, and high expression), no survival association was found. The lack of association of CD24 expression with overall survival is probably due the low number of samples with survival information.

Our results were controversial when we compared the association of CD24 with overall survival or progression free survival in the previous published paper and the data from international databases (GEPIA and Protein Atlas). This difference probably occurred because the previous literature investigates CD24 expression by immunohistochemistry (protein level) and the survival and progression-free survival data from GEPIA and Protein Atlas were evaluated at the transcriptome level (mRNA). Thus, the absence of association is due to the different techniques applied. Since immunohistochemistry evaluates CD24 protein expression and RNA-seq detects *CD24* gene expression, it is not uncommon to find dissimilarities between gene and protein expression [28]. Several molecular mechanisms can interfere at the mRNA level when a specific gene is translated into the correspondent protein, thus making it difficult to find an association between gene and protein expression and a particular phenotype [29]. 

This can explain why previous publications have demonstrated a prognostic significance of CD24 immunoexpression, while the evaluation of CD24 transcript levels did not correlate with survival. Although not associated with the overall survival, CD24 is overexpressed in cancerous prostates compared to normal prostates, as suggested by the analysis of the THPA data. This overexpression may be associated with other characteristics of the tumor that warrant further investigations and bring an interesting predictive perspective. Different therapies targeting CD24 have been proposed recently and the overexpression of CD24 in prostate cancer, as demonstrated in our study, further supports their applicability [15,16].

Several factors can be associated with mRNA dysregulation. An alteration in a gene level does not always reflect a protein modification. Thus, the lack of association of the CD24 transcript with overall survival and progression-free disease can only reflect several processes that can affect gene expression and make it difficult to associate the gene expression with clinical data. On the other hand, it seems that CD24 immunohistochemistry can be associated with clinical parameters. Consequently, a future study comparing the CD24 gene and protein expression with the same set of samples with overall survival and progression free disease would be interesting.

Regarding the role of CD24 in human prostate cancer, two previous studies have identified an association between CD24 expression and serum prostatic specific antigen (PSA) relapse [6,8]. After surgery, PSA levels in the serum are commonly decreased, while, with tumor recurrence, a PSA biochemical recurrence may be expected [30]. Thus, CD24 expression could predict which patients may have tendencies to biochemical recurrence and possible recrudescence of the tumor.

Among the studies evaluating the expression of CD24 in animal models of prostate cancer, Cremers et al. [7] unsuccessfully attempted to confirm the role of CD24 in the initial transformation and growth of prostatic neoplastic cells in a mouse model with PC. In our study, we have confirmed the overexpression of CD24 in canine prostate cancer—similar to human PC and the mouse model with PC–and though the correlation between CD24 expression and prognosis was not statistically significant, we found a tendency to lower survival rates in canine patients with positive CD24 expression. Interestingly, we identified CD24 expression on both canine cell lines (PC1 and PC2) and also the human PC3 cell line. On the Human Proteome Atlas, CD24 transcripts are also reported in human PC3 cell line. Therefore, future studies investigating the role of CD24 expression on prostate cancer can use these cellular systems to understand the role of this molecule in different cellular processes.

Among models of prostate cancer, mice and dogs have been widely used [4,31,32,33]. Usui et al. [31] established an organoid model of canine prostate cancer for new therapeutic approaches. However, no further studies evaluated the reliability of this model in predicting the anti-tumor response of different drugs. Although mice models have been used for human prostate cancer [34], dogs develop prostate cancer naturally, thus resembling human disease [32]. Several studies have evaluated the efficacy of different anti-cancer drugs in canine models of human cancers [35,36,37], particularly immune therapy strategies for canine osteosarcomas [35], use of monoclonal antibodies in canine oral melanomas [36] and use of sorafenib in hepatocellular carcinomas [37]. Taking advantage of the use of dogs in preclinical therapeutic studies, our findings may open up interesting opportunities to validate the role of CD24 as a therapeutic target for prostate cancer and further investigate how CD24 overexpression may be related to tumor features that may be useful for a specific prognostic or predictive approach.

Due the possibility of using dogs as druggable models for prostate cancer, we performed a homology analysis of human and canine CD24 gene and protein. The high homology found indicates that monoclonal antibodies and tyrosine kinase receptors developed to identify human CD24 also can identify canine CD24. Ashihara et al. [12] evaluated the pharmacokinetics of a liposomal nanoparticle encapsulated with cisplatin targeted to CD24-positive cells in a xerographic mice model. Since dogs present CD24-positive tumors and CD24 protein have a high protein homology between species, CD24-positive canine prostate cancer can be a valuable model to evaluate the clinical efficiency of this new therapy.

## 5. Conclusions

Although some previous studies demonstrated the role of CD24 immunoexpression as a prognostic factor in human prostate cancer, *CD24* gene expression performed in this study did not validate this hypothesis. Canine prostate cancer expressing CD24 may represent a potential comparative perspective to study the role of CD24 as prognostic and predictive factor in both species.

## Figures and Tables

**Table 1:** Information on and data obtained from the 10 selected manuscripts included in the systematic review.

Reference	Manuscript Goal	Samples	Methodology Used for CD24 Analysis	Number of Prostate Samples	Main Conclusions
Cremers et al. [7]	Investigate CD24 role in tumorigenesis in murine breast and prostate cancer models	Murine mammary glands, prostate, and seminal vesicles	Immunohistochemistry	52	CD24 loss has no significant effect on cancer initiation
Petkova et al. [23]	Distinguish basal subsets of human prostate cancer at different stages of differentiation using specific surface markers	Single cell suspensions of prostate tissue	Color flow cytometry	49	CD24 is a suitable marker to identify subsets at different levels of differentiation within human benign and cancerous prostate epithelium
Zhang et al. [24]	Identify inheritable genetic factors to predict PC aggressiveness	Human tumor Biopsies	Immunohistochemistry	590	There is a significant association of certain CD24 alleles to PC onset and progression
Zhang et al. [25]	Better understanding of the mechanism and significance of CD24-dependent inactivation of mutant p53 in prostate cancer cells	FFPE tissue, frozen tissue, and cell lines	Immunohistochemistry (FFPE and frozen tissue), RT-qPCR (cell lines)	543 FFPE, 21 frozen samples, and 3 cell lines	In human PC, there is a CD24-dependent inactivation of mutant p53
Wang et al. [26]	Identify a link between CD24 overexpression and functional inactivation of the tumor suppressor genes TP53 and ARF	Cell lines	Immunofluorescence, western blot, flow cytometry	3	CD24 overexpression decreases the expression of ARF and p53
Nagy et al. [8]	Measure the expression of CD24, c-MYC, and phospholipase 2a in prostate cancer tissues	Tumor Biopsies	RT-qPCR	20 PC and 11 BPH	Overexpression of CD24 is most likely associated with serum PSA levels and Gleason’s grades
Liu et al. [9]	Understanding the clinical relevance of CD24-dependent inactivation of mutant p53	FFPE tissue	Immunohistochemistry	288	CD24-p53 axis may contribute to aggressive and metastatic prostate cancers
Kristiansen et al. [20]	Identify differentially expressed genes that might be useful diagnostic or therapeutic biomarkers of prostate cancer	Frozen and FFPE prostate tissue	RT-qPCR, mRNA in situ hybridization and immunohistochemistry	91	Combined marker analysis using MEMD and CD24 expression allows improved prediction of patient prognosis
Kristiansen et al. [6]	Evaluate the status of CD24 protein expression and investigate its association with clinicopathological parameters and progression-free survival	FFPE tissue	Immunohistochemistry	31 nodal metastases and 102 PC	CD24 expression is a predictor of PSA relapse and poor prognosis in low grade or organ confined PC
Schostak et al. [27]	Evaluate the usefulness of real-time RT-qPCR for the specific and sensitive detection of *CD24* transcripts	Frozen prostate tissues	RT-qPCR	59	The quantitative *CD24* RNA transcript detection in prostatic tissues is feasible even without a previous laser microdissection

**Table 2:** Association of the clinical and pathological data with the CD24 expression pattern.

Clinico-Pathological Feature	CD24 Expression Pattern	
	Negative	Positive
Total cases	13	27
Mean age	9.3 ± 1.8	11.5 ± 1.6
**Metastasis**		
Yes	*n* = 5	*n* = 9
No	*n* = 8	*n* = 16
Histological pattern *		
Cribriform	*n* = 3	*n* = 11
Solid	*n* = 3	*n* = 6
Papillary	*n* = 2	*n* = 1
Small acinar	*n* = 5	*n* = 9
**Gleason score**		
6	*n* = 4	*n* = 6
7	*n* = 0	*n* = 1
8	*n* = 3	*n* = 4
9	*n* = 1	*n* = 2
10	*n* = 5	*n* = 14
Survival (Days)	307.7 ± 150.6	192 ± 132.6

* Main histological subtype when mixed lesions were present (primary pattern).

## Data Availability

Data sharing is not applicable to this article since all data is available in the manuscript text.

## References

[1] Jaggupilli A., Elkord E. (2012). Significance of CD44 and CD24 as Cancer Stem Cell Markers: An Enduring Ambiguity. Clin. Dev. Immunol..

[2] Deng X., Apple S., Zhao H., Song J., Lee M., Luo W., Wu X., Chung D., Pietras R.J., Chang H.R. (2017). CD24 Expression and differential resistance to chemotherapy in triple-negative breast cancer. Oncotarget.

[3] Fonseca-Alves C.E., Kobayashi P.E., Calderón L.G.R., Felisbino S.L., Rinaldi J.D.C., Drigo S.A., Rogatto S.R., Laufer-Amorim R. (2018). Immunohistochemical panel to characterize canine prostate carcinomas according to aberrant p63 expression. PLoS ONE.

[4] Costa C.D., Justo A.A., Kobayashi P.E., Story M.M., Palmieri C., Amorim R.L., Fonseca-Alves C.E. (2019). Characterization of OCT3/4, Nestin, NANOG, CD44 and CD24 as stem cell markers in canine prostate cancer. Int. J. Biochem. Cell Biol..

[5] Uhlén M., Fagerberg L., Hallström B.M., Lindskog C., Oksvold P., Mardinoglu A., Sivertsson Å., Kampf C., Sjöstedt E., Asplund A. (2015). Tissue-based map of the human proteome. Science.

[6] Kristiansen G., Pilarsky C., Pervan J., Stürzebecher B., Stephan C., Jung K., Loening S., Rosenthal A., Dietel M. (2003). CD24 expression is a significant predictor of PSA relapse and poor prognosis in low grade or organ confined prostate cancer. Prostate.

[7] Cremers N., Neeb A., Uhle T., Dimmler A., Rothley M., Allgayer H., Fodde R., Sleeman J.P., Thiele W. (2016). CD24 Is Not Required for Tumor Initiation and Growth in Murine Breast and Prostate Cancer Models. PLoS ONE.

[8] Nagy B., Szendrői A., Romics I. (2008). Overexpression of CD24, c-myc and Phospholipase 2A in Prostate Cancer Tissue Samples Obtained by Needle Biopsy. Pathol. Oncol. Res..

[9] Liu W., Zhang Y., Wei S., Bae S., Yang W., Smith G.J., Mohler J.L., DrPH E.T.H.F., Bensen J.T., Sonpavde G.P. (2020). A CD24-p53 axis contributes to African American prostate cancer disparities. Prostate.

[10] Barkal A.A., Brewer R.E., Markovic M., Kowarsky M., Barkal S.A., Zaro B.W., Krishnan V., Hatakeyama J., Dorigo O., Barkal L.J. (2019). CD24 signalling through macrophage Siglec-10 is a target for cancer immunotherapy. Nat. Cell Biol..

[11] Eyvazi S., Kazemi B., Dastmalchi S., Bandehpour M. (2018). Involvement of CD24 in Multiple Cancer Related Pathways Makes It an Interesting New Target for Cancer Therapy. Curr. Cancer Drug Targets.

[12] Ashihara K., Terai Y., Tanaka T., Tanaka Y., Fujiwara S., Maeda K., Tunetoh S., Sasaki H., Hayashi M., Ohmichi M. (2020). Pharmacokinetic evaluation and antitumor potency of liposomal nanoparticle encapsulated cisplatin targeted to CD24-positive cells in ovarian cancer. Oncol. Lett..

[13] Sun F., Wang Y., Luo X., Ma Z., Xu Y., Zhang X., Lv T., Zhang Y., Wang M., Huang Z. (2019). Anti-CD24 antibody-nitric oxide conjugate (ANC) selectively and potently suppresses hepatic carcinoma. Cancer Res..

[14] Chen Z., Wang T., Tu X., Xie W., He H., Wang M., Zhang J. (2017). Antibody-based targeting of CD24 enhances antitumor effect of cetuximab via attenuating phosphorylation of Src/STAT3. Biomed. Pharmacother..

[15] Ogilvie L.A., Kovachev A., Wierling C., Lange B.M.H., Lehrach H. (2017). Models of Models: A Translational Route for Cancer Treatment and Drug Development. Front. Oncol..

[16] Comandante-Lou N., Fallahi-Sichani M., Wolkenhauer O. (2021). Models of Cancer Drug Discovery and Response to Therapy. Systems Medicine: Integrative, Qualitative and Computational Approaches.

[17] Palmieri C., Foster R., Grieco V., Fonseca-Alves C., Wood G., Culp W., Escobar H.M., De Marzo A., Laufer-Amorim R. (2019). Histopathological Terminology Standards for the Reporting of Prostatic Epithelial Lesions in Dogs. J. Comp. Pathol..

[18] Tang Z., Li C., Kang B., Gao G., Li C., Zhang Z. (2017). GEPIA: A web server for cancer and normal gene expression profiling and interactive analyses. Nucleic Acids Res..

[19] Gambim V.V., Laufer-Amorim R., Alves R.H.F., Grieco V., Fonseca-Alves C.E. (2020). A Comparative Meta-Analysis and in silico Analysis of Differentially Expressed Genes and Proteins in Canine and Human Bladder Cancer. Front. Vet. Sci..

[20] Kristiansen G., Pilarsky C., Wissmann C., Kaiser S., Bruemmendorf T., Roepcke S., Dahl E., Hinzmann B., Specht T., Pervan J. (2004). Expression profiling of microdissected matched prostate cancer samples reveals CD166/MEMD and CD24 as new prognostic markers for patient survival. J. Pathol..

[21] Madden T. (2013). The BLAST sequence analysis tool. The NCBI Handbook [Internet].

[22] Waterhouse A., Bertoni M., Bienert S., Studer G., Tauriello G., Gumienny R., Heer F.T., de Beer T.A.P., Rempfer C., Bordoli L. (2018). SWISS-MODEL: Homology modelling of protein structures and complexes. Nucleic Acids Res..

[23] Petkova N., Hennenlotter J., Sobiesiak M., Todenhöfer T.W., Scharpf M., Stenzl A., Bühring H.-J., Schwentner C. (2013). Surface CD24 distinguishes between low differentiated and transit-amplifying cells in the basal layer of human prostate. Prostate.

[24] Zhang Y., Li B., Zhang X., Sonpavde G.P., Jiao K., Zhang A., Zhang G., Sun M., Chu C., Li F. (2017). CD24 is a genetic modifier for risk and progression of prostate cancer. Mol. Carcinog..

[25] Zhang W., Yi B., Wang C., Chen D., Bae S., Wei S., Guo R.-J., Lu C., Nguyen L.L., Yang W.-H. (2016). Silencing of CD24 Enhances the PRIMA-1–Induced Restoration of Mutant p53 in Prostate Cancer Cells. Clin. Cancer Res..

[26] Wang L., Liu R., Ye P., Wong C., Chen G.-Y., Zhou P., Sakabe K., Zheng X., Wu W., Zhang P. (2015). Intracellular CD24 disrupts the ARF–NPM interaction and enables mutational and viral oncogene-mediated p53 inactivation. Nat. Commun..

[27] Schostak M., Krause H., Miller K., Schrader M., Weikert S., Christoph F., Kempkensteffen C., Kollermann J. (2006). Quantitative real-time RT-PCR of CD24 mRNA in the detection of prostate cancer. BMC Urol..

[28] Liu Y., Beyer A., Aebersold R. (2016). On the Dependency of Cellular Protein Levels on mRNA Abundance. Cell.

[29] Koussounadis A., Langdon S.P., Um I.H., Harrison D.J., Smith V.A. (2015). Relationship between differentially expressed mRNA and mRNA-protein correlations in a xenograft model system. Sci. Rep..

[30] Krause B.J., Souvatzoglou M., Tuncel M., Herrmann K., Buck A.K., Praus C., Schuster T., Geinitz H., Treiber U., Schwaiger M. (2008). The detection rate of [11C]Choline-PET/CT depends on the serum PSA-value in patients with biochemical recurrence of prostate cancer. Eur. J. Nucl. Med. Mol. Imaging.

[31] Usui T., Sakurai M., Nishikawa S., Umata K., Nemoto Y., Haraguchi T., Itamoto K., Mizuno T., Noguchi S., Mori T. (2017). Establishment of a dog primary prostate cancer organoid using the urine cancer stem cells. Cancer Sci..

[32] Laufer-Amorim R., Fonseca-Alves C.E., Villacis R.A.R., Linde S.A.D., Carvalho M., Larsen S.J., Marchi F.A., Rogatto S.R. (2019). Comprehensive Genomic Profiling of Androgen-Receptor-Negative Canine Prostate Cancer. Int. J. Mol. Sci..

[33] Calderón L.G.R., Kobayashi P.E., Vasconcelos R.O., Fonseca-Alves C.E., Laufer-Amorim R. (2019). Characterization of Collagen Fibers (I, III, IV) and Elastin of Normal and Neoplastic Canine Prostatic Tissues. Vet. Sci..

[34] Laakso H., Ylä-Herttuala E., Sierra A., Jambor I., Poutanen M., Liljenbäck H., Virtanen H., Merisaari H., Aronen H., Minn H. (2021). Docetaxel chemotherapy response in PC3 prostate cancer mouse model detected by rotating frame relaxations and water diffusion. NMR Biomed..

[35] Mason N.J., Gnanandarajah J.S., Engiles J.B., Gray F., Laughlin D., Gaurnier-Hausser A., Wallecha A., Huebner M., Paterson Y. (2016). Immunotherapy with a HER2-Targeting Listeria Induces HER2-Specific Immunity and Demonstrates Potential Therapeutic Effects in a Phase I Trial in Canine Osteosarcoma. Clin. Cancer Res..

[36] Kamoto S., Shinada M., Kato D., Yoshimoto S., Ikeda N., Tsuboi M., Yoshitake R., Eto S., Hashimoto Y., Takahashi Y. (2020). Phase I/II Clinical Trial of the Anti-Podoplanin Monoclonal Antibody Therapy in Dogs with Malignant Melanoma. Cells.

[37] Marconato L., Sabattini S., Marisi G., Rossi F., Leone V.F., Casadei-Gardini A. (2020). Sorafenib for the Treatment of Unresectable Hepatocellular Carcinoma: Preliminary Toxicity and Activity Data in Dogs. Cancers.

